# Intravenous iRGD‐Guided, RBC‐Membrane Camouflaged *Lactococcus Lactis* Remodels Cold NSCLC and Enhances PD‐1 Blockade

**DOI:** 10.1002/advs.202509604

**Published:** 2025-10-03

**Authors:** Chen Chen, Junmeng Zhu, Xiao Liu, Jie Shao, Aoxing Chen, Yi Mei, Xinyin Zhang, Qinyi Chen, Lin Li, Baorui Liu

**Affiliations:** ^1^ Department of Oncology Nanjing Drum Tower Hospital, Affiliated Hospital of Medical School Nanjing University Nanjing 210008 China; ^2^ Department of Oncology The Affiliated Cancer Hospital of Nanjing Medical University & Jiangsu Cancer Hospital & Jiangsu Institute of Cancer Research Nanjing Jiangsu China; ^3^ Department of Oncology Nanjing Drum Tower Hospital Clinical College of Nanjing University of Chinese Medicine Nanjing 210008 China; ^4^ The Comprehensive Cancer Centre China Pharmaceutical University Nanjing Drum Tower Hospital Nanjing 210008 China; ^5^ Department of Pathology Nanjing Drum Tower Hospital Affiliated Hospital of Medical School Nanjing University Nanjing 210008 China

**Keywords:** engineered probiotics, immunotherapy resistance, non‐small cell lung cancer, PD‐1 immune checkpoint blockade, RBC membrane coating, tumor microenvironment, tumor‐penetrating peptide (iRGD)

## Abstract

Resistance to programmed‐death‐1/programmed‐death‐ligand‐1 (PD‐1/PD‐L1) blockade in non‐small‐cell lung cancer (NSCLC) arises mainly from weak tumor immunogenicity and limited effector T‐cell infiltration. Here, this work presents an intravenously deliverable “living medicine” that addresses these barriers through biomimetic cloaking, tumor‐penetrating guidance, and synthetic‐biology‐driven cytokine release. *Lactococcus lactis* is engineered to co‐secrete Flt3L and OX40L (FOLactis) and then camouflage with red‐blood‐cell membranes, producing long‐circulating mRBC@FOLactis. Conjugation of the iRGD peptide (iRGD‐mRBC@FOLactis) enables trans‐endothelial migration and deep (≥200 µm) interstitial penetration, yielding a fourfold increase in intratumorally bacterial accumulation versus unmodified FOLactis. In the orthotopic Lewis lung carcinoma (LLC) model, a single intravenous dose of iRGD‐mRBC@FOLactis combined with anti‐PD‐1 antibody achieves complete tumor regression in 60% of mice, doubles median survival (*p* < 0.001), and generates systemic tumor‐specific immune memory. Mechanistically, local Flt3L and OX40L secretion expands cross‐presenting dendritic cells (DCs), boosts CD8⁺ T‐cell priming, and converts immunologically “cold” tumors into inflamed, T‐cell‐rich lesions, thereby overcoming primary resistance to checkpoint blockade. This multifunctional probiotic platform establishes a generalizable strategy for systemic delivery of living therapeutics and offers a powerful adjunct to PD‐1/PD‐L1 blockade for NSCLC and other treatment‐resistant solid tumors.

## Graphical abstract

This study presents an engineered probiotic platform, iRGD‐mRBC@FOLactis, combining red blood cell membrane cloaking and tumor‐penetrating peptide iRGD for enhanced PD‐1 blockade. The platform improves tumor targeting, immune cell activation and therapeutic efficacy in lung cancer, demonstrating significant promise in overcoming PD‐1/PD‐L1 therapy resistance. Created with BioRender.com.

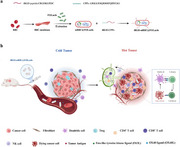

## 1 Introduction

Non‐small‐cell‐lung cancer (NSCLC) ranks among the leading malignancies worldwide in both incidence and mortality.^[^ ^1^ ^]^ Immune checkpoint blockade (ICB) targeting the PD‐1/PD‐L1 axis has reshaped NSCLC treatment,^[^ ^2^, ^3^, ^4^, ^5^, ^6^ ^]^ yet only approximately 30% of patients achieve durable clinical benefit due to primary or acquired resistance.^[^ ^7^, ^8^, ^9^ ^]^ Key mechanisms include weak tumor immunogenicity, impaired antigen presentation, and limited effector T‐cell infiltration into the tumor microenvironment (TME).^[^ ^10^, ^11^, ^12^, ^13^, ^14^, ^15^ ^]^ Thus, strategies that reprogram the immunosuppressive TME to promote T‐cell‐mediated tumor rejection are urgently needed.

In situ tumor vaccination via intratumoral delivery of immunostimulatory agents represents one such approach, enhancing antigen presentation and activating tumor‐infiltrating lymphocytes.^[^ ^16^, ^17^, ^18^ ^]^ Live bacterial therapies are a promising class of in situ vaccines, benefiting from intrinsic tumor‐homing capacity, pathogen‐associated molecular patterns (PAMPs), and genetic tractability.^[^ ^19^, ^20^, ^21^ ^]^ We previously engineered a probiotic *Lactococcus lactis* strain (“FOLactis”) to stably coexpress two potent immunostimulatory molecules: Fms‐like tyrosine kinase ligand (Flt3L) and OX40 ligand (OX40L). Intratumoral FOLactis recruited dendritic cells (DCs), enhanced antigen cross‐presentation in draining lymph nodes (TDLNs), activated NK and effector T cells, and suppressed regulatory T cells (Tregs). This generated strong systemic antitumor activity in poorly infiltrated or PD‐1/PD‐L1‐resistant tumor models, synergizing with PD‐1/PD‐L1 inhibitors.^[^ ^22^ ^]^

To address challenges of intratumoral injection—such as high interstitial pressure and dense stroma—we encapsulated FOLactis in a thermosensitive Poloxamer 407 hydrogel. This sustained‐release system improved tumor retention, boosted DC activation, and expanded memory T‐cell formation, amplifying systemic antitumor immunity.^[^ ^23^ ^]^ In malignant pleural effusion (MPE), optimized intrapleural FOLactis delivery reduced pleural tumor burden and enhanced tumor‐specific immunity via activation of CD103⁺ DCs and effector T cells.^[^ ^24^ ^]^ To overcome the limitations of personalized neoantigen peptide vaccines, we further developed Ag‐FOLactis using Plug‐and‐Display technology, enabling rapid neoantigen peptide presentation on the bacterial surface. This platform elicited potent neoantigen‐specific T‐cell responses with epitope spreading, achieving therapeutic efficacy in both subcutaneous and metastatic tumor models.^[^ ^25^ ^]^

However, intratumoral or intralymphatic injections restrict use to accessible lesions, underscoring the need for systemic delivery platforms to treat disseminated or deep‐seated tumors. Prior systemic bacterial therapies faced rapid clearance and poor tumor targeting; for instance, intravenous Salmonella (VNP20009) showed limited tumor colonization and dose‐limiting toxicities, leading to clinical trial termination.^[^ ^26^, ^27^ ^]^ To overcome these barriers, we cloaked FOLactis with red blood cell (RBC) membranes, which display “self” markers such as CD47 that suppress macrophage uptake.^[^ ^28^ ^]^ RBC membrane coating, known to prolong nanoparticle circulation,^[^ ^29^, ^30^ ^]^ also extended bacterial half‐life 14‐fold and increased tumor accumulation 42‐fold while reducing systemic inflammation.^[^ ^31^ ^]^​ Based on these findings, we developed RBC‐coated FOLactis (mRBC@FOLactis) via membrane extrusion, hypothesizing that this biomimetic disguise would enable immune evasion and efficient tumor delivery.

To further enhance tumor homing and penetration, we functionalized mRBC@FOLactis with the tumor‐penetrating cyclic peptide iRGD (CRGDKGPDC). iRGD binds αvβ3/β5 integrins on tumor vasculature and, upon proteolytic cleavage, interacts with neuropilin‐1 to trigger deep tissue penetration.^[^ ^32^, ^33^ ^]^ Incorporating iRGD onto RBC membranes via lipid insertion yielded iRGD‐mRBC@FOLactis, a biomimetic bacterial platform with superior tumor‐targeting capacity.

**Scheme 1:**
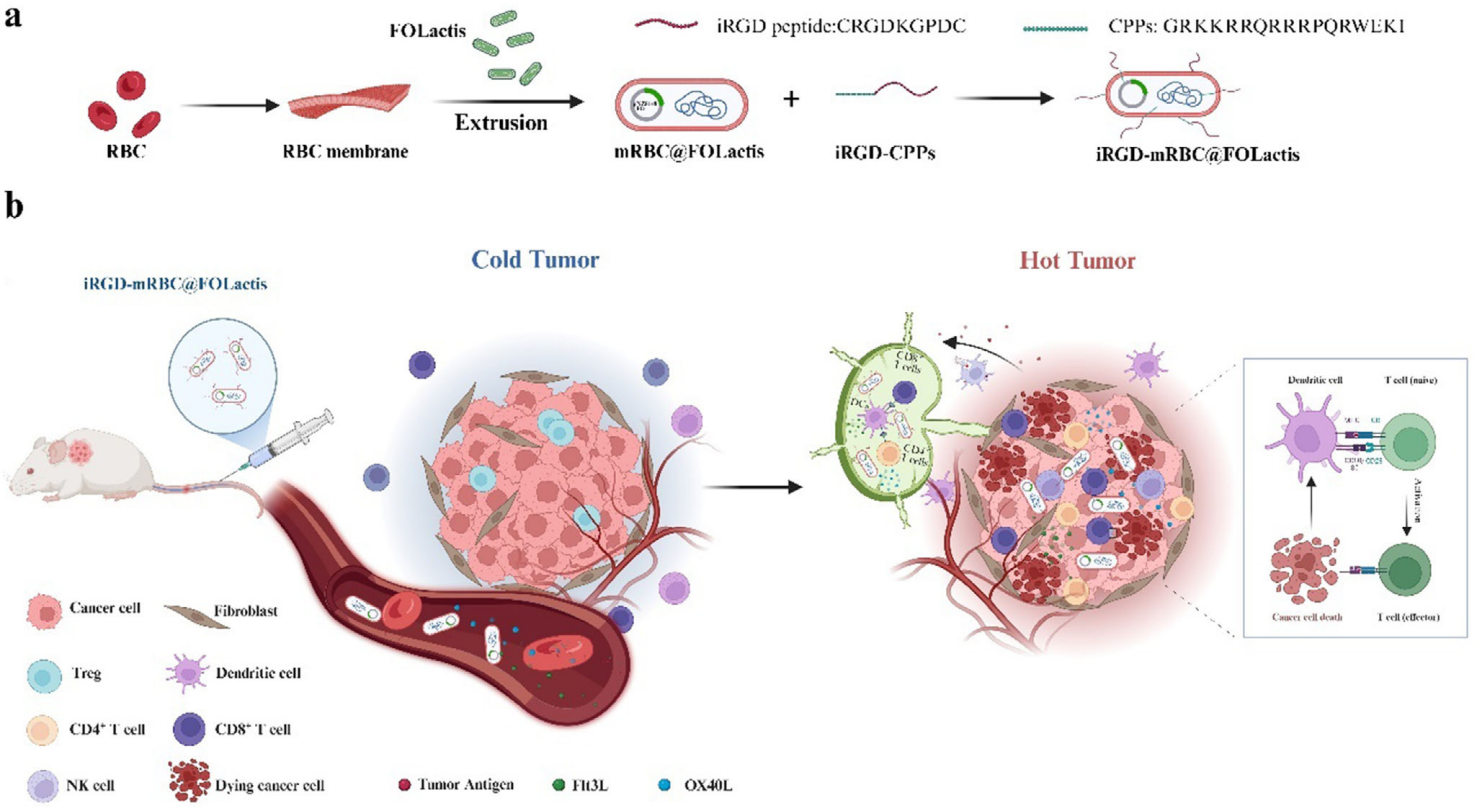
Schematic illustration depicting the construction and immunotherapeutic mechanism of iRGD‐mRBC@FOLactis. a) Preparation of iRGD‐mRBC@FOLactis by cloaking engineered FOLactis bacteria with red blood cell membranes and further functionalizing with tumor‐penetrating peptide iRGD via cell‐penetrating peptides (CPPs). b) Intravenously administered iRGD‐mRBC@FOLactis transforms immunologically "cold" tumors characterized by impaired dendritic cell (DC) activation and limited T‐cell infiltration into "hot" tumors. This transformation enhances DC recruitment, promotes antigen presentation, facilitates effector T‐cell infiltration and activation, suppresses immunosuppressive cells (e.g., Tregs), and ultimately improves response to anti‐PD‐1 immunotherapy. Created with BioRender.com; no statistical analysis performed.

We hypothesize that intravenous iRGD‐mRBC@FOLactis can effectively target and penetrate NSCLC tumors, reprogram the TME, and potentiate PD‐1 blockade. Flt3L and OX40L secretion by FOLactis is expected to expand DCs and activate T cells within tumors, converting “cold” tumors into “hot” immune‐infiltrated lesions. By combining RBC cloaking and iRGD targeting, this systemically deliverable platform overcomes the limitations of local therapies. In this study, we evaluate the therapeutic efficacy and underlying immune mechanisms of iRGD‐mRBC@FOLactis in combination with PD‐1 blockade in a mouse lung cancer model. This approach holds substantial clinical promise for improving the tumor immune landscape in NSCLC and other solid tumors, while reversing both primary and acquired resistance to PD‐1/PD‐L1 therapies (**Scheme** **1**).

## 2 Results

### 2.1 Preparation and Characterization of mRBC@FOLactis

**Figure 1:**
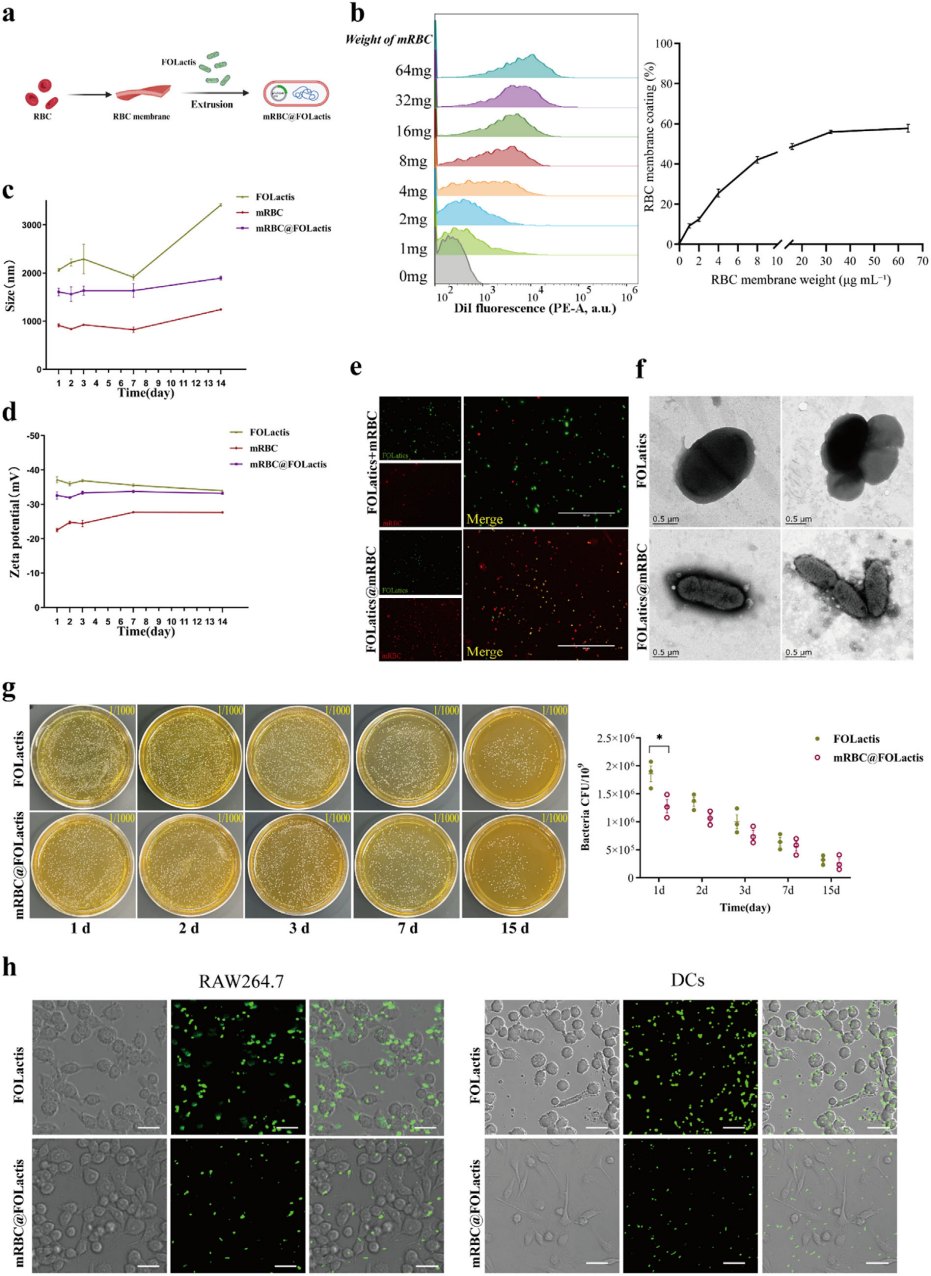
Preparation and characterization of mRBC@FOLactis. a) Schematic illustration of the preparation process for mRBC@FOLactis. Red blood cells (RBCs) were isolated and lysed to extract RBC membranes, which were then mixed with engineered Lactis (FOLactis) cells and processed through mechanical extrusion. b) Flow‐cytometry histograms of DiI‐labeled, RBC‐membrane–coated FOLactis at increasing RBC membrane input (0–64 mg per 1 × 10^9^ CFU), and quantification of DiI‐positive bacteria (%). Data are mean ± SD, *n* = 3 independent preparations; one‐way ANOVA with Tukey's post‐hoc test. c) Hydrodynamic diameter measurements of mRBC@FOLactis obtained by dynamic light scattering (DLS) at different time points. d) Zeta potential measurements of mRBC@FOLactis at different RBC membrane concentrations. e) Fluorescence microscopy images of mRBC@FOLactis. FOLactis inherently expressed GFP (green), and RBC membranes were labeled with Dil (red). Merged images show RBC membrane coverage on FOLactis cells. Scale bar = 20 µm. f) Transmission electron microscopy (TEM) images showing RBC membranes surrounding FOLactis cells. g) Colony formation of mRBC@FOLactis on agar plates. Representative images of colonies after plating FOLactis and mRBC@FOLactis. h) Fluorescence and bright‐field microscopy images showing the uptake of mRBC@FOLactis (green) by RAW264.7 macrophages and dendritic cells (DCs) after 6 h of incubation. Scale bar = 50 µm. Data are presented as mean ± standard deviation (SD). Statistical differences were analyzed using one‐way ANOVA followed by Tukey's post hoc test. Representative images in e–h; no statistical analysis performed. ^*^p < 0.05, ^**^p < 0.01, ^***^p < 0.001.

Engineered mRBC@FOLactis were prepared by isolating RBCs from mice, extracting their membranes, and fusing these membranes with FOLactis through mechanical extrusion (**Figure** **1**a). To optimize coating efficiency, different amounts of RBC membrane (2–64 mg per 1 × 10⁹ bacteria) were assessed by flow cytometry. Increasing membrane input progressively enhanced membrane association, with saturation observed at 32 mg, which was identified as the optimal dosage (Figure 1b). Flow cytometry confirmed successful RBC membrane coating of FOLactis. Staining with anti‐CD47 showed a dose‐dependent increase in CD47⁺ bacterial events, reaching a plateau at ≈32 µg mL^−1^ of membrane proteins, consistent with the DiI labeling results (Figure S1a,b, Supporting Information). After coating, bacterial surfaces were examined by confocal fluorescence microscopy. In these experiments, FOLactis expressed GFP intrinsically, while RBC membranes were pre‐labeled with DiI. The merged images showed distinct colocalization of green and red fluorescence, confirming uniform membrane coverage (Figure 1e). Transmission electron microscopy (TEM) provided complementary structural insights, revealing that each bacterium was fully encapsulated by a continuous, homogeneous, and intact membrane layer (Figure 1f). Dynamic light scattering (DLS) further demonstrated stable hydrodynamic diameters of mRBC@FOLactis over time, indicating excellent colloidal stability (Figure 1c). Zeta potential analysis corroborated these results, showing increasingly negative surface charges with greater membrane input, eventually approximating that of native RBCs (Figure 1d). Colony formation assays on GM17 agar confirmed that membrane coating did not impair bacterial viability or growth over a 15‐day culture period (Figure 1g).

To assess the functional impact of RBC membrane camouflage on immune evasion, an in vitro phagocytosis assay was performed. Murine RAW264.7 macrophages and DCs were cocultured at 37 °C with either GFP‐expressing FOLactis (uncoated) or mRBC@FOLactis for 6 h. After thorough PBS washing to remove extracellular bacteria, cells were imaged under standardized conditions using confocal fluorescence microscopy. The images revealed markedly weaker GFP signals in cells exposed to mRBC@FOLactis compared to uncoated FOLactis, demonstrating that RBC membrane camouflage effectively reduces bacterial uptake by immune cells (Figure 1h).

### 2.2 In Vivo Evaluation of mRBC@FOLactis: Antitumor Efficacy and Safety

**Figure 2:**
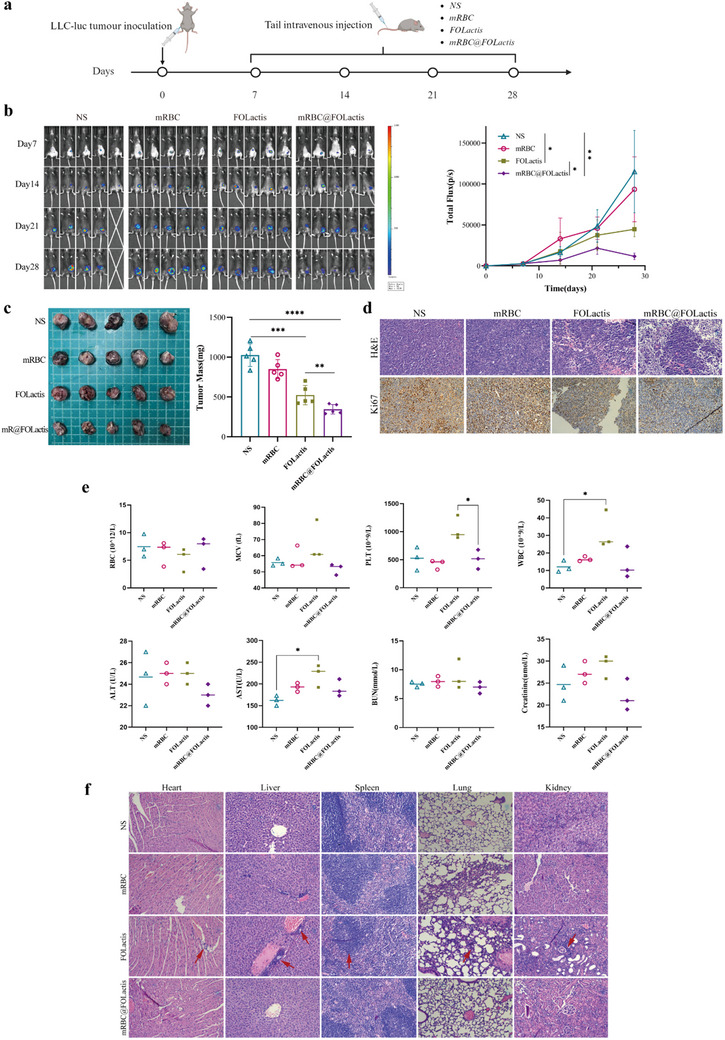
Evaluation of biocompatibility, safety, and antitumor effects of intravenously injected mRBC@FOLactis in LLC tumor‐bearing mice. a) Schematic illustration of subcutaneous LLC tumor establishment and intravenous injection of mRBC@FOLactis in C57BL/6 mice. b) Representative near‑infrared fluorescence images of LLC tumor‑bearing mice at the indicated time points following intravenous injection of NS, mRBC, FOLactis, or mRBC@FOLactis, together with the corresponding fluorescence intensity–time curves for each treatment group. c) Ex vivo tumor weights at the study endpoint for each treatment group (NS, mRBC, FOLactis, mRBC@FOLactis). d) Representative images of tumor tissues stained with H&E (top) and Ki67 immunohistochemistry (bottom) from different groups at the study endpoint. Scale bars as indicated. e) Blood routine and serum biochemical indices at study endpoint, including white blood cell (WBC), red blood cell (RBC), platelet (PLT) counts, alanine aminotransferase (ALT), aspartate aminotransferase (AST), and other biochemical parameters among different groups. f) Representative histological sections (H&E staining) of major organs (heart, liver, spleen, lung, kidney) from different treatment groups at the study endpoint. Scale bar = 100 µm. Data are presented as mean ± SD, *n* = 5. Statistical differences were analyzed by one‐way ANOVA followed by Tukey's multiple comparisons test; Panels d and f are representative images; no statistical analysis performed. ^*^p < 0.05, ^**^p < 0.01, ^***^p < 0.001.

In our in vivo study, we established a subcutaneous tumor model using LLC‐Luc cells, which stably expressed luciferase, for monitoring tumor progression after treatment. The mice received intravenous injections of different formulations, and at predetermined time points (i.e., 7, 14, 21, and 28 days), they were administered D‐luciferin intraperitoneally, following which they were subjected to bioluminescence imaging by using an IVIS Spectrum system. Considering that tumor cells emit light via the luciferase activity, the measured photon flux served as an indirect indicator of tumor growth. Notably, the tumors in the mRBC@FOLactis‐treated group displayed significantly lower bioluminescence over the observation period when compared to those in the NS, mRBC, and uncoated FOLactis groups (**Figure** **2**a,b), suggesting an effective inhibition of tumor progression.

At the end of the study, the tumors were excised and weighed ex vivo. The tumor masses in the mRBC@FOLactis group were found to be significantly lower than those in the control groups, and the differences were highly statistically significant (*p* < 0.0001; Figure 2c). Histopathological analysis by H&E staining revealed that the tumor tissues from mice treated with mRBC@FOLactis exhibited a loosely arranged architecture, with extensive necrosis and marked inflammatory infiltration, whereas the tumors in the other groups maintained a densely cellular structure with minimal necrosis. Consistently, Ki67 immunohistochemistry demonstrated a significant reduction in the proportion of proliferating cells in the mRBC@FOLactis group (Figure 2d).

Further safety assessments were conducted through routine blood tests and serum biochemistry. The mRBC@FOLactis group showed significantly lower white blood cell counts, platelet counts, and AST levels compared to the uncoated FOLactis group, while other indicators—including RBC count, mean corpuscular volume (MCV), ALT, BUN, and creatinine—remained comparable across groups (Figure 2e). Histological examination of major organs (heart, liver, spleen, lung, and kidney) using H&E staining revealed no pathological abnormalities in mRBC@FOLactis‐treated mice. By contrast, although the NS and mRBC groups displayed no notable changes, the uncoated FOLactis group exhibited marked inflammatory cell infiltration in the heart, liver, lung, and kidney (highlighted by red arrows) (Figure 2f). These findings indicate that RBC membrane coating substantially enhances the biocompatibility of engineered bacteria and mitigates inflammatory responses, thereby ensuring systemic safety. Collectively, these data confirm that intravenous administration of mRBC@FOLactis not only suppresses tumor growth effectively but also maintains excellent systemic safety and biocompatibility.

### 2.3 Enhanced Tumor Penetration by iRGD Functionalization

**Figure 3:**
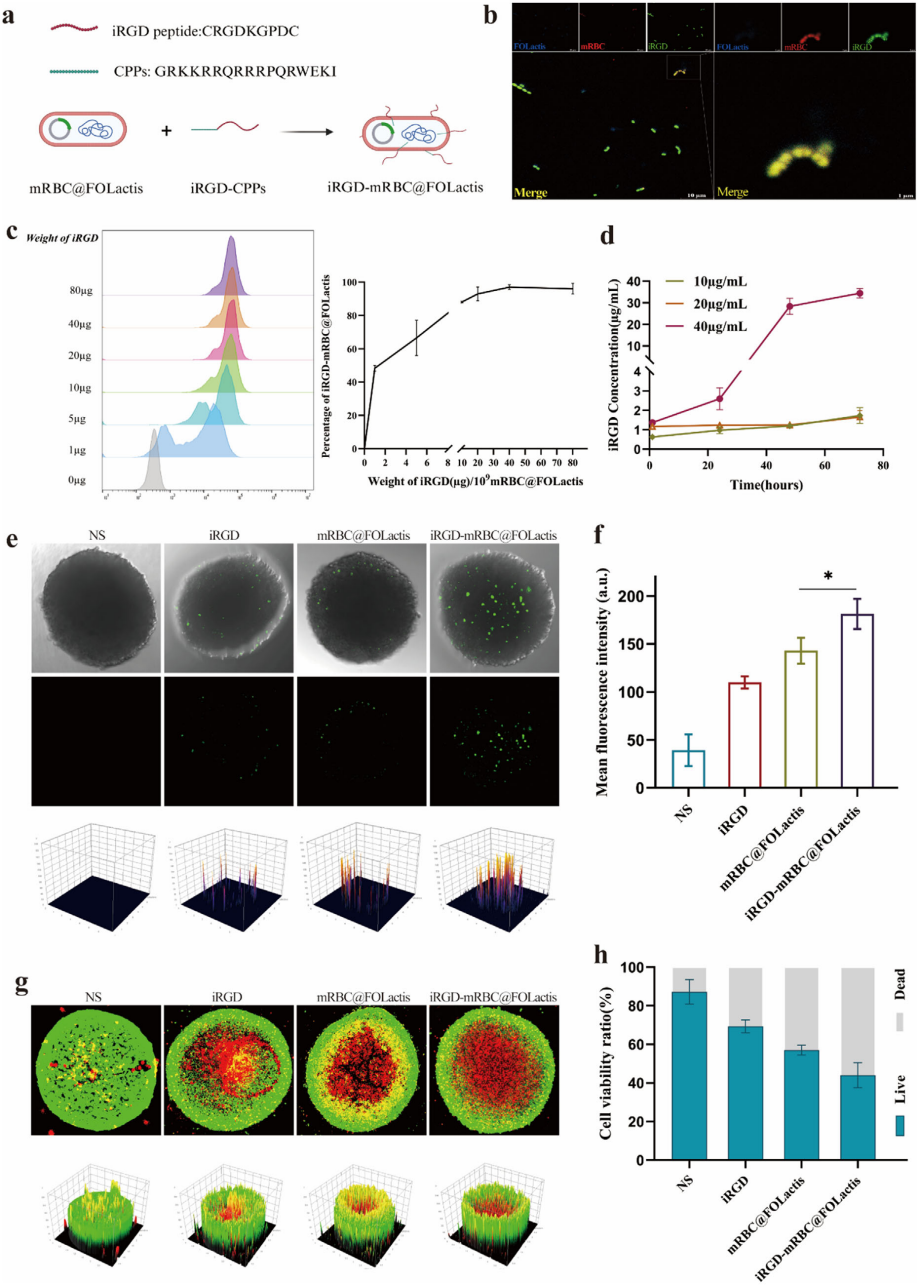
Construction and application of iRGD‐modified mRBC@FOLactis for targeted drug delivery and tumor penetration. a) Schematic illustration of iRGD‐modified mRBC@FOLactis construction. b) Confocal microscopy of iRGD‐modified mRBC@FOLactis. Confocal microscopy images of iRGD‐modified mRBC@FOLactis. DAPI (blue) labels FOLactis cells, Dil (red) labels the mRBC membrane, and iRGD peptides are labeled green. The merged image shows the successful modification of mRBC@FOLactis with iRGD peptide. c) Flow cytometry analysis of iRGD peptide modification efficiency. Flow cytometry histograms show the fluorescence intensity of mRBC@FOLactis modified with iRGD peptides at different peptide‐to‐particle ratios (per 10^9^ mRBC@FOLactis). d) Quantification of unbound iRGD peptide in the supernatant by HPLC after modification. e) 3D tumor spheroid penetration assay. 3D tumor spheroids were treated with the following four groups: (1) NS (control), (2) iRGD, (3) mRBC@FOLactis, and (4) iRGD‐mRBC@FOLactis. Fluorescent microscopy images show the extent of drug penetration into the tumor spheroids. f) Quantification of drug penetration in 3D tumor spheroids. g) Cell viability assay (live/dead staining). Cell viability was assessed using live/dead staining. Dead cells are stained red, and live cells are stained green. Fluorescent images show the effects of the treatments on tumor cells. h) Quantification of cell viability from live/dead staining. Data are mean ± SD, *n* = 3; one‐way ANOVA with Tukey's post‐hoc test. Panels b, e, g are representative images; no statistical analysis performed. ^*^p < 0.05, ^**^p < 0.01.

To enhance tumor penetration and therapeutic efficacy, mRBC@FOLactis was functionalized with the tumor‐penetrating peptide iRGD through a cell‐penetrating peptide (CPP) mediated lipid‐insertion strategy (**Figure** **3**a). Flow cytometry was performed to determine the optimal peptide loading onto mRBC@FOLactis, displaying a dose‐dependent increase in the fluorescence intensity correlating with higher 5‐FAM‐iRGD‐CPPs concentrations. At lower peptide concentrations (≤5 µg per 1 × 10⁹ bacteria), the fluorescence signal was modest, whereas a significant enhancement occurred at 10–20 µg concentration (*p* < 0.01), and saturation was reached at 40 µg, with no further increment recorded at 80 µg, indicating maximal conjugation at 40 µg (Figure 3c).

The conjugation efficiency of the iRGD peptide to mRBC@FOLactis was further validated by analyzing free peptide concentrations in the reaction supernatant through high‐performance liquid chromatography (HPLC). At peptide loading concentrations of 10 and 20 µg mL^−1^, minimal free peptide was detected over the 72‐h analysis period, confirming efficient membrane conjugation. In contrast, at the highest tested concentration (40 µg mL^−1^), a noticeable accumulation of unbound peptide occurred over time, implying that the membrane's binding capacity had been exceeded (Figure 3d). To evaluate the stability of iRGD functionalization, dual‐color flow cytometry was performed using CD47–AF647 and FAM‐labeled iRGD. A high proportion of dual‐positive (FAM⁺/CD47⁺) events was observed at baseline and remained detectable after up to 15 days of serum incubation, indicating that both the RBC membrane and the iRGD peptide were stably retained on the bacterial surface (Figure S2a, Supporting Information).

Confocal fluorescence microscopy provided visual confirmation of successful peptide conjugation. The merged images demonstrated the colocalization of DAPI‐stained FOLactis bacteria (blue), DiI‐labeled RBC membranes (red), and 5‐FAM‐labeled iRGD peptides (green), which presented a uniform distribution across the bacterial surface. High‐magnification images further confirmed robust and homogeneous colocalization, indicative of effective and stable peptide incorporation through the CPP‐mediated lipid insertion method (Figure 3b).

The enhanced tumor‐penetration capability of iRGD‐mRBC@FOLactis was further assessed by using an A549 cell‐derived three‐dimensional (3D) tumor spheroid model. After a 24‐h treatment, the spheroids in the NS control group exhibited minimal green fluorescence, indicating negligible bacterial penetration. In the free iRGD group, green fluorescence was weakly detectable only at the spheroid periphery. Spheroids treated with unmodified mRBC@FOLactis displayed a small amount of fluorescence predominantly at the outer layers, whereas those treated with iRGD‐mRBC@FOLactis exhibited extensive and uniform green fluorescence throughout the spheroid, demonstrating significantly improved penetration (Figure 3e). Quantitative analysis confirmed that the fluorescence intensity in the iRGD‐mRBC@FOLactis group was significantly higher relative to that in the other groups (*p* < 0.01; Figure 3f).

The cytotoxic effects of iRGD‐mRBC@FOLactis were evaluated through live/dead staining using Calcein‐AM and propidium iodide. Spheroids treated with NS maintained high viability (>85%), whereas those treated with free iRGD exhibited a modest reduction (approximately 70% viability). In contrast, spheroids exposed to unmodified mRBC@FOLactis demonstrated a noticeable decrease in cell viability (approximately 60%, *p* < 0.05), while the iRGD‐mRBC@FOLactis group exhibited the most pronounced cytotoxic effect, with viability falling to approximately 40% (*p* < 0.01 compared to all other groups) (Figure 3g,h). These results collectively indicated that the combination of RBC membrane coating and iRGD functionalization significantly improved the penetration and cytotoxicity of mRBC@FOLactis, thereby offering a promising approach for enhanced tumor targeting.

### 2.4 In Vivo Distribution of iRGD‐mRBC@FOLactis

**Figure 4:**
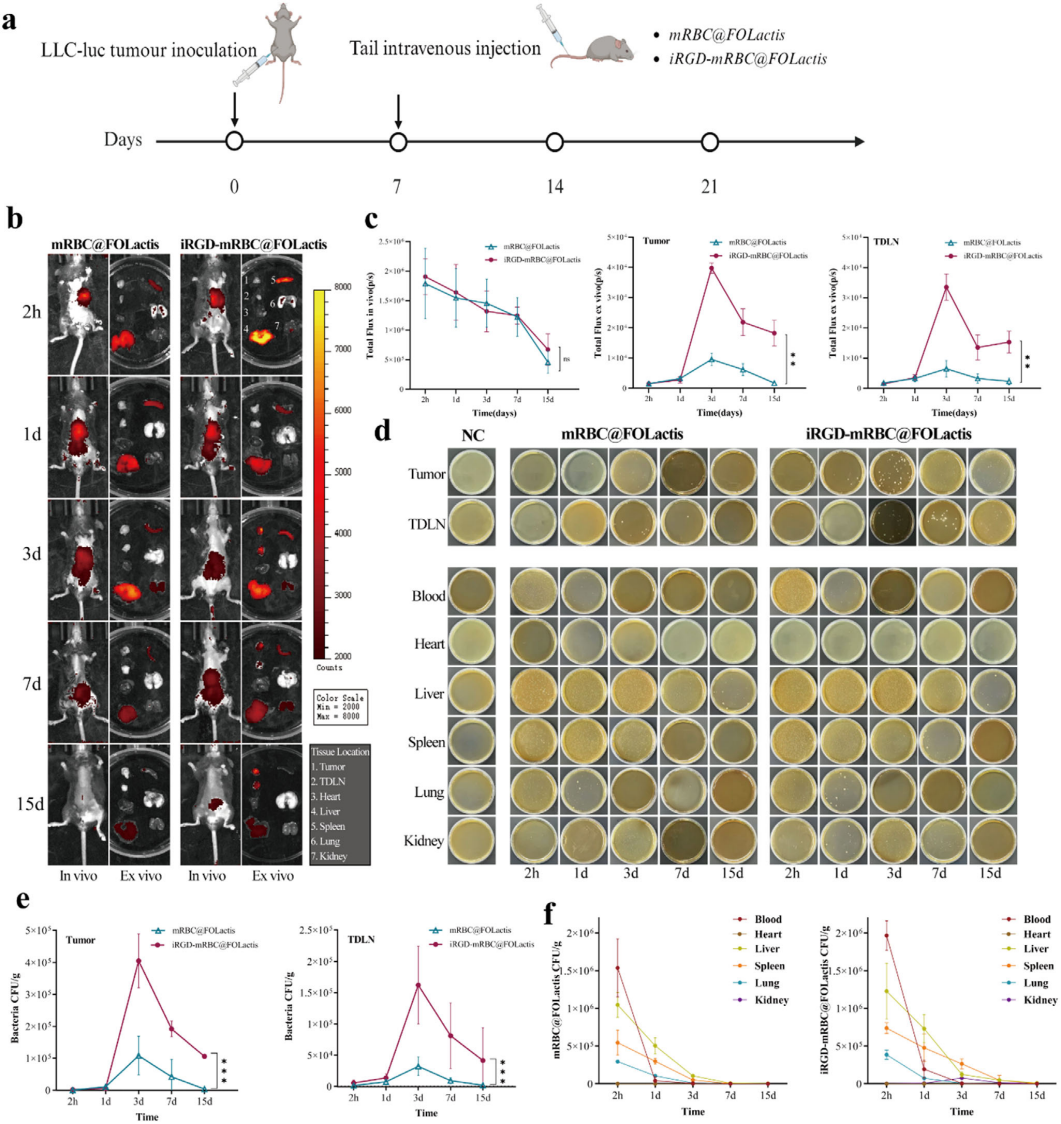
Distribution of intravenously injected iRGD‐mRBC@FOLactis in mice. a) Schematic illustration: Subcutaneous injection of LLC‐luc cells was performed to establish a tumor model in C57BL/6 mice. The mice were randomly divided into two groups: the mRBC@FOLactis group and the iRGD‐mRBC@FOLactis group. The drugs were administered via tail vein injection. b) In vivo fluorescence imaging for observing the distribution in tumors and major organs: Near‐infrared fluorescence imaging was used to monitor the distribution of DiR‐labeled mRBC@FOLactis and iRGD‐mRBC@FOLactis in tumors and major organs at 2 h, 1 day, 3 days, 7 days, and 15 days post‐injection. c) Quantitative data analysis: It shows the quantitative data of fluorescence intensity at different time points. d–f) Bacterial plating analysis of tissue samples: Major organ and tumor tissue samples were collected for bacterial plating and counting to determine the number of bacteria in each tissue at different time points. Data are mean ± SD, *n* = 5; one‐way ANOVA with Tukey's post‐hoc test. Panel b shows representative images; no statistical analysis was performed. ^*^p < 0.05, ^**^p < 0.01.

In vivo biodistribution of the formulations was examined by using an NSCLC mouse model established through a subcutaneous inoculation of LLC cells. The mice were randomly assigned to receive intravenous injections of either iRGD‐mRBC@FOLactis or unmodified mRBC@FOLactis (**Figure** **4**a). Near‐infrared (NIR) imaging was performed at multiple time points (2 h, 24 h, 3 days, 7 days, and 15 days post‐injection) to monitor the distribution of the carriers. At 2 h post‐injection, the iRGD‐mRBC@FOLactis group exhibited a strong fluorescence signal at the tumor site that gradually increased, reaching a peak at 3 days, and then slowly diminished. However, at every time point, the tumor fluorescence in the iRGD‐modified group was significantly higher compared to that observed in the unmodified group (*p* < 0.05; Figure 4b,c). In addition, imaging of the excised tumors confirmed that the iRGD‐mRBC@FOLactis‐treated tumors maintained a higher fluorescence intensity compared to those treated with unmodified mRBC@FOLactis. Lymph node imaging also revealed enhanced fluorescence in the TDLNs of the iRGD‐modified group, suggesting that iRGD facilitates not only tumor targeting but also migration to the regional lymphatics. In contrast, fluorescence signals in the liver, spleen, lungs, and kidneys were minimal, indicating the preferential accumulation of the carriers in the tumors and lymphatic tissues, while sparing the non‐target organs, exhibiting favorable biocompatibility.

Ex vivo colony‐forming unit (CFU) analysis further quantified the distribution of viable bacteria. At early time points (2 h to 1‐day post‐injection), both groups displayed a relatively low CFU in the tumor tissues. By Day 3, however, the tumors from the iRGD‐mRBC@FOLactis group contained approximately three times more viable bacteria compared to those from the unmodified group (*p* < 0.01; Figure 4d). Although the CFU in tumors decreased gradually from Days 7 to 14, the iRGD‐mRBC@FOLactis group maintained significantly higher bacterial counts. A similar trend was observed in the TDLNs, where the bacterial CFU peaked on Day 3 and, despite a gradual decline, remained significantly high compared to the unmodified group (*p* < 0.01). In contrast, while both the groups initially demonstrated high bacterial counts in the blood and major organs (notably in the liver and spleen) within 2 h to 1 day, these counts dropped rapidly by days 7 to 15, and by day 15, the bacterial count in the non‐target tissues was negligible, with no significant differences between the groups (Figure 4e,f).

Collectively, these imaging and CFU results indicate that iRGD modification markedly enhanced the active targeting and retention of mRBC@FOLactis in the tumor tissues and TDLNs to facilitate rapid clearance of the tumor from the healthy organs. This dual functionality underscores the potential of iRGD‐mRBC@FOLactis as a safe and effective platform for targeted tumor therapy.

### 2.5 Synergistic Antitumor Effect of iRGD‐mRBC@FOLactis and PD‐1 Blockade

**Figure 5:**
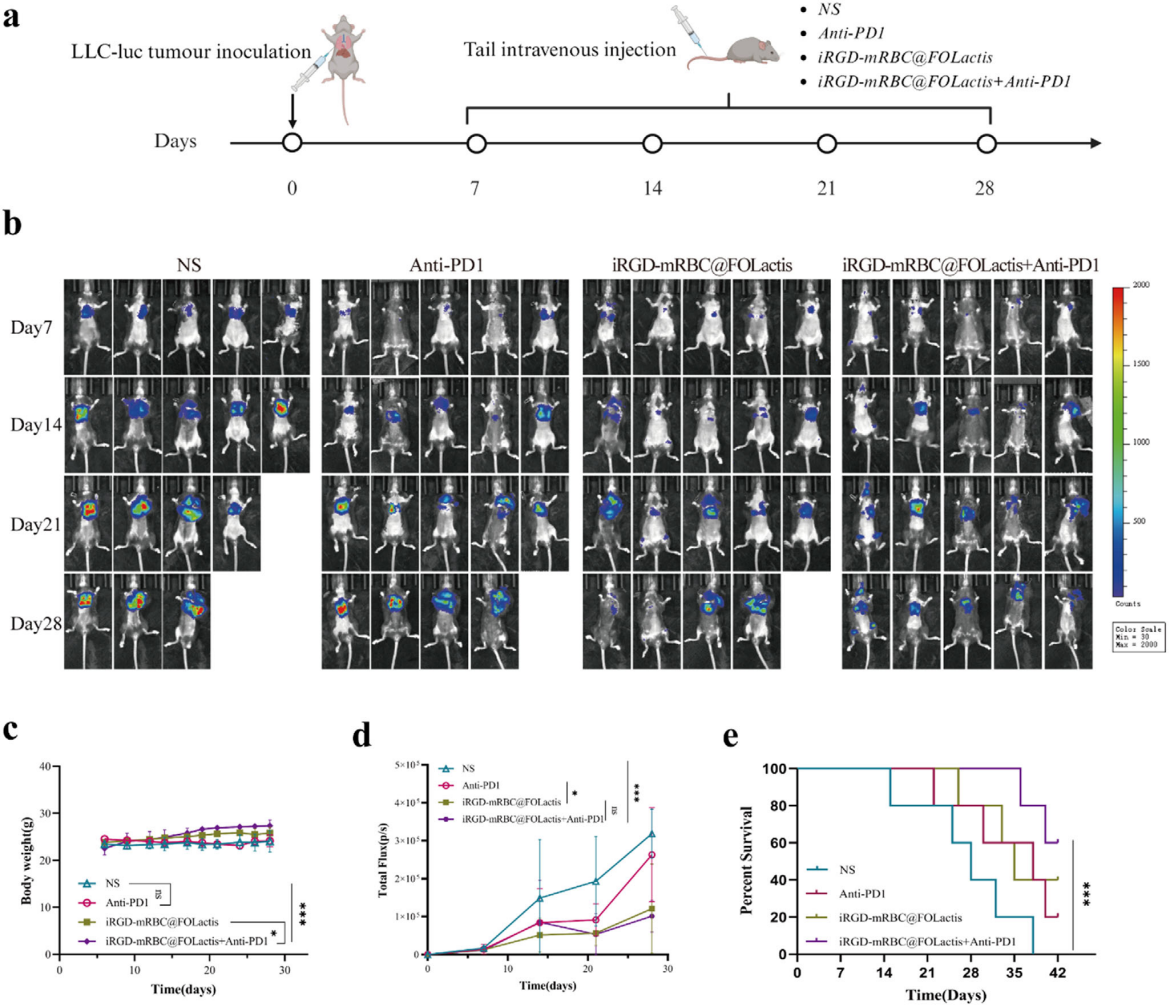
Treatment of lung cancer in an orthotopic lung cancer mouse model with iRGD‐mRBC@FOLactis combined with PD‐1 antibody. a) Experimental design and treatment groups: LLC cells were implanted into C57BL/6 mice to establish an orthotopic lung cancer model. Mice were randomly assigned to four groups: NS (control), PD‐1 antibody, iRGD‐mRBC@FOLactis, and PD‐1 antibody + iRGD‐mRBC@FOLactis (either combined or sequential treatment). Treatments were administered via tail vein injection. b,d) Near‐infrared imaging for tumor growth assessment: Tumor growth was monitored using a near‐infrared imaging system. Images were captured at multiple time points to assess the tumor progression in different treatment groups. Mean ± SD, *n* = 5; one‐way ANOVA with Tukey's post‐hoc test. c) Body weight changes of mice: The body weight of mice was measured throughout the study. Data show the body weight changes across different treatment groups. Mean ± SD, *n* = 5; no significant differences. e) Survival analysis of mice: The survival rate of mice in each treatment group was monitored throughout the study. Kaplan–Meier survival curves, log‐rank test, *n* = 6 mice per group. ^*^p < 0.05, ^**^p < 0.01, ^***^p < 0.001.

In this study, we evaluated the therapeutic efficacy and safety of combining iRGD‐mRBC@FOLactis with PD‐1 antibody in an orthotopic lung cancer model. For this purpose, C57BL/6 mice were implanted with LLC cells to establish a lung cancer model. The mice were then randomly assigned to one of the following 4 groups: NS control, PD‐1 antibody monotherapy, iRGD‐mRBC@FOLactis monotherapy, and a combination group, which received both PD‐1 antibody and iRGD‐mRBC@FOLactis via tail vein injection (**Figure** **5**a). This experimental design was intended to comparatively assess the efficacy of the combination therapy and individual treatments. Using dynamic monitoring with NIR fluorescence imaging, we observed that the tumors in the control group (NS) grew the fastest, showing increasing fluorescence signals over time. The monotherapy groups, treated with either PD‐1 antibody or iRGD‐mRBC@FOLactis alone, exhibited some tumor growth inhibition. However, their tumor size continued to increase. In contrast, the combination therapy group (iRGD‐mRBC@FOLactis + PD‐1 antibody) exhibited the weakest fluorescence signals and the smallest tumor size, indicating the most significant suppression of the tumor growth. The quantitative analysis of the total fluorescence intensity revealed that the control group displayed a continuous increase in fluorescence, which was significantly higher in this group compared to that in the other groups. Both the PD‐1 antibody and iRGD‐mRBC@FOLactis monotherapy groups demonstrated some reduction in the fluorescence intensity, although the combination group demonstrated the lowest fluorescence intensity, indicating the most effective tumor suppression (Figure 5b). Statistical analysis confirmed that the combination therapy group had a significantly greater tumor inhibition effect when compared to the monotherapy groups and the control group (*p* < 0.05, Figure 5d).

Regarding the impact of the treatments on mouse health, the body weight remained stable across all groups, showing no significant differences, suggesting that the treatments did not adversely affect the overall health of the mice (Figure 5c). In terms of survival, the control group (NS) exhibited the shortest survival times, with a higher mortality rate. The mice treated with PD‐1 antibody alone or iRGD‐mRBC@FOLactis alone showed a slight extension in their survival time, although the difference was not significant. In contrast, the combination therapy group significantly outperformed the monotherapy groups, with a significantly higher survival rate (*p* < 0.001, Figure 5e). The median survival of the combination group was nearly double that of the control groups, indicating that the combination therapy not only effectively inhibits tumor growth but also significantly enhances their survival.

In conclusion, combining iRGD‐mRBC@FOLactis with PD‐1 antibody achieved strong antitumor efficacy in an orthotopic NSCLC mouse model. This strategy markedly suppressed tumor growth, prolonged survival, and showed no adverse effects on overall health. These results highlight the promising clinical potential of this combination therapy for treating NSCLC.

### 2.6 Immunomodulatory Effects of Combination Therapy on the TME

**Figure 6:**
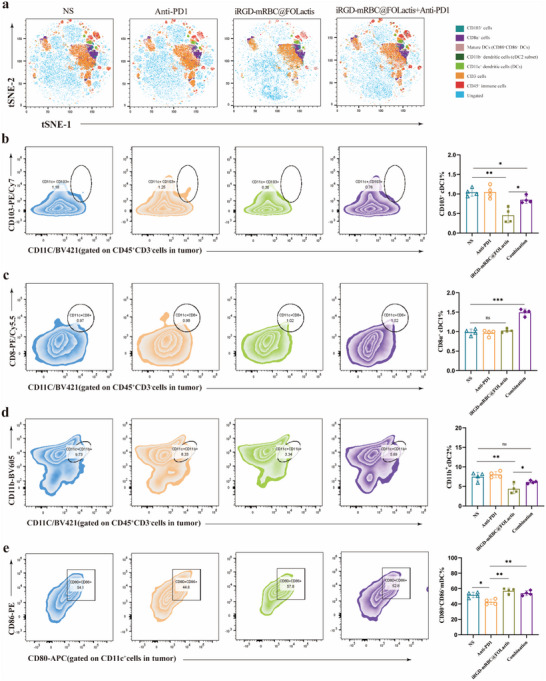
Flow cytometric analysis of dendritic cell (DC) subsets in a murine orthotopic lung cancer model. a) t‑SNE plots: CD45⁺ immune cells isolated from tumors were visualized by t‑SNE to display the distribution of leukocyte subpopulations under each treatment; no statistical analysis performed. b) CD103⁺ DCs: Representative FACS plots and quantification of CD103⁺ DCs (gated as CD45⁺CD3^−^CD11c⁺) in tumors, comparing their frequency across the four groups. c) CD8α⁺ DCs: Representative FACS plots and quantification of CD8α⁺ DCs (gated as CD45⁺CD3^−^CD11c⁺) in tumors, showing changes among the treatment arms. d) CD11b⁺ DCs: Representative FACS plots and quantification of CD11b⁺ DCs (gated as CD45⁺CD3^−^CD11c⁺) in tumors, illustrating the relative abundance in each group. e) Mature DCs (CD80⁺CD86⁺): Representative FACS plots and quantification of co‑stimulatory marker expression on CD11c⁺ DCs (gated as CD11c⁺) in tumors, reflecting maturation status across treatments. All bar graphs show mean ± SEM. Statistical comparisons were made by one‑way ANOVA with Tukey's post hoc test; ^*^p < 0.05, ^**^p < 0.01, ^***^p < 0.001.

**Figure 7:**
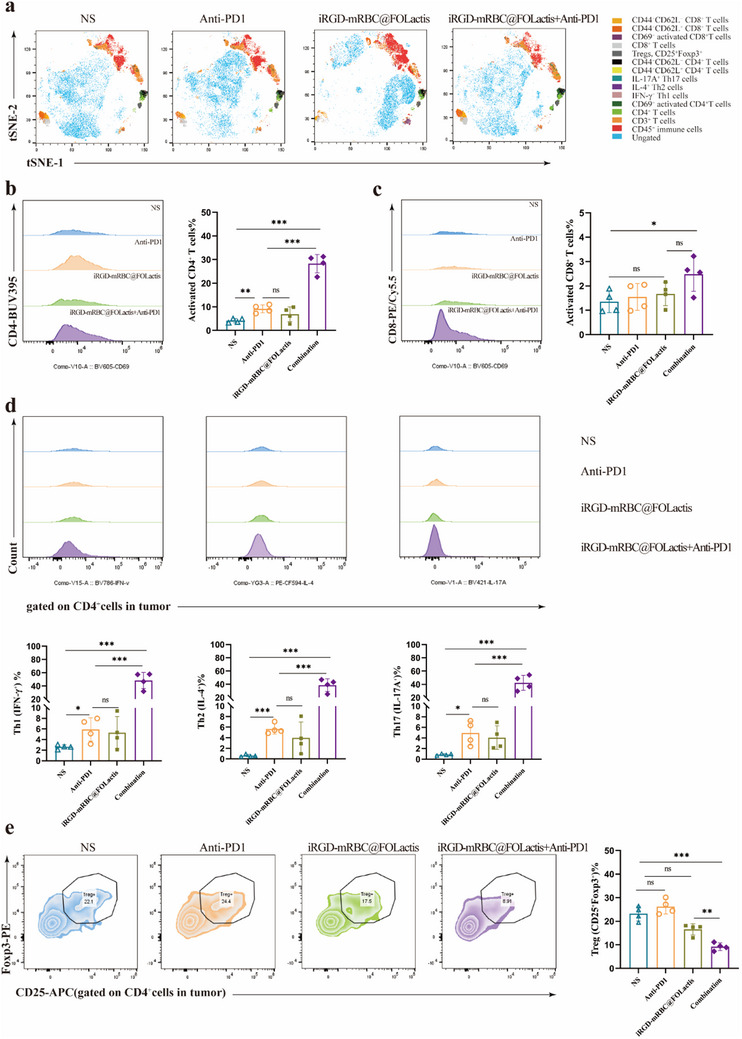
Flow cytometric analysis of T‐cell subsets in a murine orthotopic lung cancer model. a) t‑SNE plots of CD45⁺ cells isolated from tumor digests, showing the distribution of major immune cell clusters in each treatment group; no statistical analysis performed. b) Representative violin plots and quantification of activated CD4⁺ T cells (CD4⁺CD69⁺ gated on CD45⁺CD3⁺), illustrating the proportion of CD4⁺ T‐cell activation under each treatment. c) Representative violin plots and quantification of activated CD8⁺ T cells (CD8⁺CD69⁺ gated on CD45⁺CD3⁺), demonstrating differences in CD8⁺ T‐cell activation among the groups. d) Representative violin plots and quantification of Th1, Th2, and Th17 subsets (IFN‑γ⁺, IL‑4⁺, and IL‑17A⁺ gated on CD4⁺), reflecting helper T‐cell polarization. e) Representative contour plots and quantification of regulatory T cells (CD25⁺Foxp3⁺ gated on CD4⁺), measuring Treg frequency in each group. All data are shown as mean ± SEM; statistical analysis by one‑way ANOVA with Tukey's post hoc test; ^*^p < 0.05, ^**^p < 0.01, ^***^p < 0.001.

Comprehensive immune profiling was performed on the excised tumor tissues to investigate how different immunotherapy strategies affect the TME. The tumor samples were processed into single‐cell suspensions, and flow cytometry was performed to delineate any changes in the immune cell populations. Unsupervised t‐SNE analysis of CD45⁺ cells revealed distinct alterations in the immune landscape among the treatment groups (**Figures** 6, 7).

Flow cytometric analysis of the tumor‐infiltrating immune cells was performed to assess the impact of the different treatment regimens on the TME. The detailed analysis of the DC subsets revealed that the proportion of CD103⁺ DC (cDC1) was the lowest in tumors from the iRGD‐mRBC@FOLactis monotherapy group, and, although the combination of iRGD‐mRBC@FOLactis with PD‐1 blockade produced a modest increase in CD103⁺ DCs, the difference compared to that in the other treatment groups was not statistically significant (Figure 6b). In contrast, the percentage of CD11b⁺ DC (cDC2) in the TME did not exhibit any advantage with iRGD‐mRBC@FOLactis monotherapy relative to that in the control and PD‐1 antibody groups. However, in the combination group, the level of CD11b⁺ DC recovered to values that were essentially comparable to those observed in the control group (Figure 6d). Notably, the proportion of CD8⁺ DCs was significantly highest in the combination treatment group (*p* < 0.001, Figure 6c), clearly indicating that the addition of PD‐1 blockade could markedly promote the enrichment of this critical subset in the tumor. Furthermore, the analysis of mature DCs, as assessed by the coexpression of CD80 and CD86, demonstrated that the PD‐1 antibody monotherapy group exhibited the lowest maturation levels, whereas the combination therapy substantially enhanced DC maturation (*p* < 0.01, Figure 6e). Overall, these observations suggested that, while iRGD‐mRBC@FOLactis alone may not significantly augment certain DC subpopulations, its combination with PD‐1 inhibition could effectively improve both the recruitment and activation of DCs, thereby contributing to a more robust antitumor immune response.

The analysis of T‐cell populations in the TME revealed that CD8⁺ T‐cell activation was lowest in the control (NS) group. Treatment with either PD‐1 antibody or iRGD‐mRBC@FOLactis alone produced modest, nonsignificant increases in CD8⁺ T‐cell activity. In contrast, the combination of iRGD‐mRBC@FOLactis with PD‐1 antibody markedly enhanced CD8⁺ T‐cell activation, reaching levels significantly higher than those in the monotherapy groups (*p* < 0.001, Figure 7c). This synergy indicates that concurrent treatment substantially promotes antitumor immune responses.

Parallel analysis of CD4⁺ T‐cell subsets showed that combination therapy significantly increased Th1 cells, characterized by IFN‐γ secretion (*p* < 0.001), thereby shifting the TME toward an antitumor phenotype. Th17 cells, defined by IL‐17A production, were also significantly elevated in the combination group (*p* < 0.001), further amplifying the immune response. Notably, Th2 cells (IL‐4⁺ CD4⁺ T cells) also showed significant differences among the treatment groups (*p* < 0.05; Figure 7d), suggesting that the therapy influenced Th2 abundance in the TME.

Additionally, Tregs (CD4⁺CD25⁺Foxp3⁺) were abundant in the control group, consistent with a strongly immunosuppressive environment. While PD‐1 antibody or iRGD‐mRBC@FOLactis monotherapy modestly reduced Treg levels, the combination therapy induced a pronounced decrease (*p* < 0.01, Figure 7e), effectively alleviating tumor‐associated immunosuppression. Collectively, these results demonstrate that the combined iRGD‐mRBC@FOLactis and PD‐1 blockade not only enhances effector T‐cell activation but also reduces inhibitory Treg activity, thereby fostering a more favorable immune environment for tumor rejection.

### 2.7 Time‐Dependent Immune Activation Induced by iRGD‐mRBC@FOLactis

**Figure 8:**
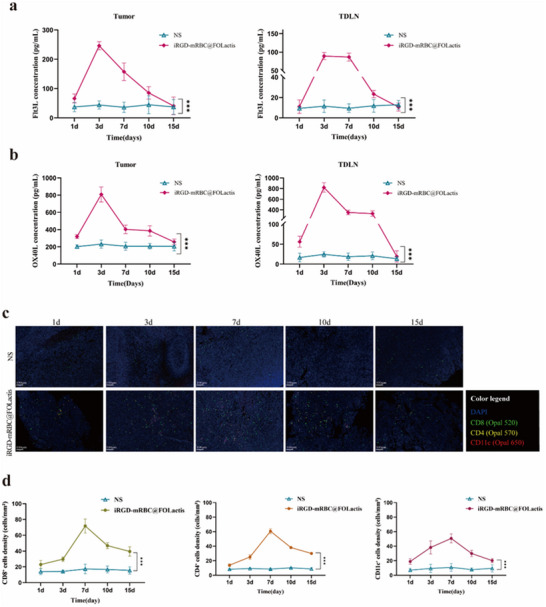
Kinetics of Flt3L and OX40L release and time‐resolved remodeling of the tumor immune microenvironment. a) ELISA quantification of Flt3L in tumor tissues (left) and tumor‐draining lymph nodes (TDLN, right) at 1, 3, 7, 10, and 15 days after treatment with iRGD‐mRBC@FOLactis or normal saline (NS). b) ELISA quantification of OX40L in tumor tissues (left) and TDLN (right) at the indicated time points. c) Representative multiplex immunofluorescence images of tumor sections collected at different time points, showing infiltration of CD8⁺ T cells (Opal 520, green), CD4⁺ T cells (Opal 570, yellow), and CD11c⁺ dendritic cells (DCs) (Opal 650, red); nuclei were counterstained with DAPI (blue). Scale bars, 200 µm. d) Quantitative analysis of multiplex immunofluorescence, depicting temporal changes in the density of CD8⁺, CD4⁺, and CD11c⁺ cells (cells mm^−^^2^) in tumors. Data are shown as mean ± SD. Statistical significance versus NS: *p* < 0.05, ^*^ *p* < 0.01, ^**^ *p* < 0.001 (two‐way ANOVA with post hoc test).

To further delineate the kinetics of immune modulation, we examined cytokine release and immune cell infiltration over time after iRGD‐mRBC@FOLactis treatment. ELISA assays demonstrated that both Flt3L and OX40L concentrations in tumors and tumor‐draining lymph nodes (TDLN) increased rapidly, peaked on Day 7, and declined by Days 10 and 15 (**Figure** **8**a,b).

Consistently, mIHC analysis revealed a parallel temporal pattern of immune remodeling. CD8⁺ T‐cell infiltration was already enhanced at Day 3, reached a maximum on Day 7, and decreased thereafter. CD11c⁺ DCs followed the same trajectory with a Day 7 peak, while CD4⁺ T‐cell recruitment exhibited a more modest increase that also waned by Day 15 (Figure 8c,d).

These findings highlight that iRGD functionalization triggers a transient but robust wave of immune activation, with maximal cytokine production and immune‐cell infiltration occurring at Day 7, followed by a gradual decline.

## 3 Discussion

Cumulatively, our findings establish the feasibility of intravenous administration of a tumor‐targeted probiotic for NSCLC therapy, in which engineered FOLactis is cloaked in autologous RBC membranes and functionalized with a tumor‐penetrating iRGD peptide. Enveloping the bacteria in autologous RBC vesicles endows them with “self” markers, such as CD47, which suppress macrophage uptake and complement activation, thereby prolonging the circulation time and reducing systemic inflammation.^[^ ^28^, ^34^, ^35^ ^]^ This phenomenon mirrors earlier studies, wherein *Escherichia coli* Nissle 1917 (EcN) was camouflaged with erythrocyte membranes to create “cell‐membrane‐coated bacteria” (CMCB). These stealth bacteria exhibited approximately 14‐fold higher blood retention at 48 h, 42‐fold greater tumor accumulation, and significantly reduced systemic inflammation when compared to uncoated EcN while maintaining bacterial viability and function.^[^ ^31^, ^36^ ^]^ Subsequent research by Alapan et al. further explored RBC membrane‐coated bacteria, such as wrapping *E. coli* MG1655 in RBC membranes loaded with doxorubicin and superparamagnetic iron oxide nanoparticles.^[^ ^37^ ^]^ This approach facilitated targeted drug delivery and enhanced therapeutic efficacy. In addition, past studies have investigated hybrid membranes, combining RBC and platelet membranes, so as to improve the circulation time and tumor homing.^[^ ^38^ ^]^ These collective findings underscore the reliability of biomembrane cloaking in imparting “self” markers to bacteria, facilitating the evasion of phagocytosis and enhancing tumor colonization without the need for genetic modification.

To further improve tumor targeting, we incorporated the cyclic iRGD peptide (CRGDKGPDC) onto mRBC@FOLactis to construct iRGD‑mRBC@FOLactis by using its dual binding to α_vβ_3/α_vβ_5 integrins and neuropilin‑1 to activate the CendR pathway.^[^ ^33^, ^39^ ^]^ This process drives deep endothelial penetration and promotes uniform distribution throughout the tumor core. Time‐course fluorescence (Figure 4b,c) and organ‐wise CFU (Figure 4d–f) jointly define retention/clearance across organs. Elimination aligns with complement‐mediated opsonization/lysis,^[^ ^40^, ^41^ ^]^ phagocytic uptake by macrophages/DCs,^[^ ^42^, ^43^, ^44^ ^]^ and NK‐cell cytotoxicity,^[^ ^45^, ^46^ ^]^ with debris handled by the reticuloendothelial system and hepatobiliary/renal excretion.^[^ ^47^, ^48^, ^49^ ^]^ Endpoint chemistry/hematology (Figure 2e), major‐organ H&E (Figure 2f), and serum IL‐6/TNF‐α support an acceptable safety window. In past studies, RGD and iRGD peptides have been commonly used in the formulations of nanoparticles and other drugs, which significantly enhanced the delivery of drugs and immune cells into tumors.^[^ ^50^, ^51^, ^52^, ^53^, ^54^ ^]^ In recent years, research on peptide display on live bacteria has shown promising outcomes in early stage tumor therapies. For instance, genetically modified *Salmonella*, displaying RGD motifs on their surface, demonstrated increased binding to α_vβ_3‐overexpressing cancer cells, resulting in the regression of integrin‐rich xenografts and extended survival in breast cancer and melanoma models.^[^ ^55^ ^]^

We previously investigated several local delivery strategies for the engineered *L. lactis* vaccine (FOLactis) expressing Flt3L and OX40L. Direct intratumoral injection of FOLactis elicits a strong local antitumor immune response, marked by an influx of conventional type‐1 DCs (cDC1) and activated tumor‐infiltrating T cells in the TME​.^[^ ^22^ ^]^ These in situ vaccination methods—including intratumoral injection, encapsulation in injectable hydrogels, intrapleural delivery, and intranodal (lymph node) injection—successfully convert the treated tumor from an immunologically “cold” state to a “hot,” inflamed state and, in some cases, even induce regression of untreated distant tumors (an abscopal effect)​.^[^ ^23^, ^24^, ^25^ ^]^ However, the antitumor activity of these local treatments remains largely restricted to the delivery sites and has a limited effect on disseminated metastatic lesions, owing to the narrow distribution and finite persistence of the therapy. As a result, tumors outside the treated area often gain only partial benefit, and some initially controlled lesions eventually recur once the in situ vaccine's influence diminishes.^[^ ^25^ ^]^

To overcome these limitations, the present study employed an intravenous delivery strategy for FOLactis, augmented with RBC membrane camouflage and iRGD peptide modification for tumor targeting. This is the first demonstration of systemically delivering FOLactis via the bloodstream to multiple tumor sites while preserving its immunotherapeutic activity, thereby surmounting the constraints of previous local‐delivery approaches. The RBC membrane coating endows the bacteria with “stealth” properties that prolong their circulation time, and the addition of the tumor‐penetrating peptide iRGD markedly enhances their accumulation and deep penetration into tumors​. Consequently, our systemic FOLactis platform effectively reached metastatic tumors and maintained robust immunostimulatory effects in vivo. Notably, this approach confers a unique advantage in globally reprogramming the TME across widespread tumor foci. We observe pronounced cDC1 recruitment in tumors throughout the body, a vigorous activation of CD8^+^ cytotoxic T cells and Th1‐type CD4^+^ T cells, and a reduction in the immunosuppressive cells such as M2‐polarized macrophages and Tregs in the tumor sites​. In other words, previously “cold” tumor lesions have uniformly turned “hot” and immunologically active, resulting in robust anti‐tumor responses even against disseminated diseases.

Recent advances in the related areas further highlight the significance of our work. For example, cell membrane coating has expanded from RBCs to platelets, cancer cells, and hybrid membranes, not only prolonging the circulation and promoting immune evasion but also serving as a multifunctional platform for targeting and immune modulation.^[^ ^56^, ^57^ ^]^ Increasing evidence suggests that hybrid and functionalized membranes can enhance tumor delivery and immune activation synergistically.^[^ ^58^ ^]^ Our group has contributed to this field by developing mRBC‐coated drug nanoparticles that improved stability and safety.^[^ ^59^, ^60^ ^]^ In addition, the application of lipid insertion to anchor iRGD‐EGFR fusion proteins on RBC membranes enhanced the delivery efficiency and tumor penetration.^[^ ^61^ ^]^ Meanwhile, iRGD applications have extended beyond conventional drug delivery to radiosensitization and immunotherapy.^[^ ^62^, ^63^ ^]^ Notably, conjugating iRGD with T cells or PD‐1 antibodies has been shown to improve tumor infiltration and local immune effects.^[^ ^64^, ^65^ ^]^ Together with our recent findings that HuFOLactis combined with iRGD enhances immune infiltration and synergizes with PD‐1 blockade,^[^ ^66^ ^]^ these studies demonstrate that iRGD is evolving from a penetration enhancer into a powerful tool for cancer immunotherapy.

Engineered bacteria are also emerging as promising “living medicines,” leveraging their tumor tropism and sustained immune‐stimulating capacity.^[^ ^67^ ^]^ We previously established a continuum of approaches in this field: FOLactis engineered to secrete Flt3L and OX40L remodeled “cold” tumors and enhanced PD‐1 blockade^[^ ^22^ ^]^; a thermosensitive hydrogel‐FOLactis system acted as an “artificial lymph island,” sustaining release and expanding memory T cells^[^ ^23^ ^]^; intrapleural administration of FOLactis suppressed malignant effusion and improved the local immune milieu^[^ ^24^ ^]^; and a Plug‐and‐Display platform enabled rapid neoantigen decoration and potent epitope spreading via intranodal delivery.^[^ ^68^ ^]^ We also demonstrated that HuFOLactis combined with iRGD amplified immune activation and synergized with PD‐1 blockade.^[^ ^66^ ^]^ Together, these studies chart a translational path from local and cavity‐based delivery to nodal vaccination, providing a solid foundation for the present systemic strategy of “mRBC camouflage + iRGD targeting + engineered *L. lactis*.” This integrated approach addresses the key challenges encountered in intravenous bacterial therapy and explains its strong synergy with PD‐1 blockade.

Compared with other emerging strategies to overcome immunotherapy resistance, our platform provides distinct advantages. Oncolytic viruses replicate within tumors and stimulate immunity but are hindered by pre‐existing neutralizing antibodies, limited genetic payloads, and the frequent need for intratumoral injection.^[^ ^69^, ^70^, ^71^ ^]^ mRNA and neoantigen vaccines elicit strong T‐cell responses but depend on individualized antigen identification and are less effective in tumors with low mutational burden.^[^ ^72^, ^73^ ^]^ Intratumoral STING or TLR agonists can turn cold tumors hot, yet their activity is short‐lived and restricted to the injected site.^[^ ^74^, ^75^, ^76^ ^]^ By contrast, our probiotic system requires no antigen discovery, secretes cytokines in situ for days, and disseminates to every lesion, functioning as a fully autonomous, self‐amplifying vaccine that addresses tumor heterogeneity and overcomes both local and metastatic disease.

Nonetheless, challenges remain in its application. Our current RBC coating covers approximately 60% of bacteria, leaving some unprotected; optimizing membrane fusion protocols and exploring hybrid membranes (e.g., RBC + cancer cell) may improve uniformity.^[^ ^77^, ^78^ ^]^ Scaling membrane preparation under GMP conditions and ensuring reproducible, stable coatings will thus be essential for clinical translation.^[^ ^79^ ^]^ Repeated dosing could induce anti‑carrier immunity; hence, incorporating genetic kill‑switches or immunomodulatory circuits could mitigate this risk.^[^ ^80^ ^]^ Finally, murine models cannot capture the full complexity of human tumors and immune regulation, warranting further studies in humanized mice and larger animals.^[^ ^81^ ^]^ In addition, broader limitations should be acknowledged. Although our platform displays promise in preclinical models, the complexity of human tumor heterogeneity and patient‐to‐patient variability presents a major translational hurdle. Manufacturing consistency, long‐term safety, and regulatory considerations will require systematic evaluation in parallel with efficacy studies. As a living bacterial therapy, balancing tumor colonization with biosafety remains a central challenge. Addressing these issues will not only strengthen the foundation for clinical translation but also guide rational improvements, such as integrating additional immunomodulatory payloads, refining delivery routes, and combining with checkpoint inhibitors or conventional modalities. In parallel with addressing these challenges, this living platform can be further armed with additional payloads (e.g., IL‑12, bispecific T‑cell engagers),^[^ ^82^, ^83^, ^84^ ^]^ combined with other checkpoint inhibitors or radiochemotherapy for additive effects, and tested in notoriously resistant cancers, such as pancreatic adenocarcinoma. By marrying biomimetic cloaking, peptide‑guided homing, and on‑site cytokine delivery, iRGD‑mRBC@FOLactis exemplifies a next‑generation “living medicine” capable of converting immunotherapy‑resistant tumors into ones that respond robustly to checkpoint blockade.

## 4 Conclusion

In summary, this biomimetic, tumor‐targeted probiotic platform illustrates a compelling strategy to overcome resistance to immune checkpoint therapy. By safely delivering live immunotherapy agents systemically, it can bridge the gap between localized in situ vaccination and whole‐body treatment. The synergy observed with PD‐1 blockade highlights how modulating the TME can unlock the full potential of T cells in an otherwise unresponsive tumor. Considering its advantages in targeting, penetration, and multifaceted immune activation, the iRGD‐mRBC@FOLactis approach holds considerable promise for improving the outcomes in NSCLC patients who do not respond to current immunotherapies. With further optimization and rigorous validation, this strategy could pave the way for a new generation of “smart” living medicines that convert immunologically cold tumors into hot, treatable ones, thus offering new hope in the fight against refractory cancers.

## 5 Experimental Section

### Study Design

The primary objective of this study was to develop a comprehensive engineered probiotic platform for personalized cancer immunotherapy. Based on our engineered FOLactis system, the strategy involves two key modifications: (1) cloaking the bacteria with RBC membranes to improve blood circulation and immune evasion, and (2) functionalizing the RBC‐coated bacteria with the tumor‐penetrating peptide iRGD to enhance tumor targeting and penetration. In vitro, the physicochemical properties of these constructs were characterized, their resistance to phagocytosis was evaluated, and their ability to penetrate 3D tumor spheroids using flow cytometry, confocal microscopy, DLS, and TEM, was measured. In vivo, the antitumor efficacy was assessed in both subcutaneous and orthotopic lung tumor models established with LLC cells. Tumor volumes, biodistribution, immune cell infiltration, and overall survival were monitored at defined time intervals. All procedures were performed in a blinded manner with at least three independent replicates, and sample sizes are indicated in the respective figure legends.

### Reagents, Bacterial Strains, Cell Lines, and Mice

The FOLactis and its fluorescent derivative FOLactis‐sfGFP were constructed in our laboratory using standard genetic engineering methods to facilitate the stable secretion of immunostimulatory molecules such as Flt3L and OX40L. These bacterial strains were routinely cultured under anaerobic conditions at 37 °C in GM17 broth and harvested during the logarithmic growth phase.

For in vitro studies, particularly for establishing three‐dimensional tumor spheroids, the A549 cell line was employed, which was cultured in RPMI 1640 medium (Gibco, #61 870 036) supplemented with 10% fetal bovine serum (Gibco, #16 170 078), 100 U mL^−1^ penicillin, and 100 µg mL^−1^ streptomycin (Meilunbio, #MA0110). Regular Mycoplasma screening was performed to ensure cell line integrity.

For in vivo tumor modeling, two cell lines were used: the LLC cells, cultured in RPMI 1640 medium as above, and the luciferase‐expressing LLC‐LUC cells, which were maintained in DMEM medium (Gibco, #11 965 092). Male or female C57BL/6 mice (5–6 weeks old, 18–22 g) were procured from Shanghai Sippr‐BK Laboratory Animal Co., Ltd. (Shanghai, China) and housed under Specific Pathogen‐Free (SPF) conditions at the Experimental Animal Center of the Affiliated Nanjing Drum Tower Hospital of Nanjing University Medical School. The facility maintained controlled conditions, including temperature (68–79 °F), humidity (30%–70%), and a 12‐h light/dark cycle (lights on between 6 am and 6 pm). Mice had free access to food and water. All animal experiments were conducted in accordance with protocols approved by the Experimental Animal Care and Use Committee of the Affiliated Nanjing Drum Tower Hospital of Nanjing University Medical School (protocol number 2024AE01105).

### Peptides

In this study, surface functionalization was achieved using a fusion peptide—specifically, iRGD‐cpps and its fluorescently labeled derivative, 5‐FAM‐iRGD‐cpps—to enhance tumor homing and penetration of the RBC membrane‐coated FOLactis bacteria. Unlike the conventional cyclic iRGD peptide (cyclo(CRGDKGPDC)), the approach employs a chimeric peptide that incorporates a cell‐penetrating peptide (CPP) moiety. The iRGD‐cpps, bearing the sequence GRKKRRQRRRPQRWEKICRGDKGPDC, serve as the tumor‐targeting domain, while the 5‐FAM‐iRGD‐cpps contain an N‐terminal CPP segment that facilitates lipid insertion into the RBC membrane as well as fluorescence tracking via the 5‐FAM label. Both peptides were synthesized at high purity (>95%) by a specialized vendor and provided in a lyophilized form. Before use, the peptides were reconstituted in sterile saline to the desired concentration. The functionalization process involved incubating the reconstituted peptides with pre‐formed RBC membrane‐coated FOLactis, allowing the CPP‐mediated lipid insertion to stably anchor the iRGD moiety onto the bacterial surface, thereby significantly enhancing tumor targeting. The selected MFC peptides were initially predicted and validated using established protocols, and all peptides were manufactured by Bankpeptide Biotechnology (Hefei, China).^[^ ^25^, ^66^, ^85^ ^]^

### RBC Membrane Coating of Bacteria

To enhance blood circulation and tumor accumulation, engineered FOLactis were cloaked with RBC membranes. Fresh whole blood (1–2 mL per mouse) was collected from healthy C57BL/6 mice via orbital sinus puncture into EDTA‐coated tubes. The blood was centrifuged at 3000 rpm for 10 min at 4 °C to remove plasma and buffy coat. The RBC pellet was washed three times with sterile normal saline and then subjected to hypotonic lysis using 0.25× PBS on ice for about 10 min to release hemoglobin and obtain RBC membrane fragments. Following a brief centrifugation (3000 rpm for 5 min), the membrane pellet was resuspended gently in PBS. For the coating process, the isolated RBC membranes were mixed with FOLactis (1 × 10^9^ CFU) on ice at a defined membrane‐to‐bacteria ratio and then extruded 20 times through an 800 nm polycarbonate membrane using a mini‐extruder, yielding RBC membrane‐coated bacteria (mRBC@FOLactis). The final formulation was maintained on ice until use.

### Flow Cytometry for Bacterial Characterization

Engineered *L. lactis* (FOLactis) was coated with RBC membranes as described. To quantify coating efficiency, RBC membranes were pre‐labeled with DiI, mixed with FOLactis at the indicated membrane‐to‐bacteria ratios, extruded, and washed to remove unbound vesicles. Samples were analyzed on a flow cytometer at 25 °C under standardized settings. Bacterial events were identified by FSC/SSC characteristics, with doublets excluded by standard area/height (A/H) discrimination. DiI fluorescence was collected in the PE channel (area signal, PE‐A) at fixed detector settings across groups. For each sample, ≥50 000 events were acquired.

Readouts included the percentage of DiI‐positive bacteria (relative to an unstained FOLactis control) and the median fluorescence intensity (MFI, a.u.) of DiI‐positive events. Controls comprised FOLactis only, DiI‐labeled RBC‐membrane vesicles only, and fluorescence‐minus‐one (FMO) controls. Data were processed in standard flow‐analysis software, and results from *n* = 3 independent preparations are reported as mean ± SD, with statistical testing performed as specified in the Statistical Analysis subsection. For consistency with the figures, the *x*‐axis for histograms is labeled “DiI fluorescence (PE‐A, a.u.)”.

To further quantify RBC membrane coating and assess iRGD retention, mRBC@FOLactis were stained with Alexa Fluor 647–conjugated anti‐CD47 at 4 °C for 30 min, washed, and analyzed. Coating efficiency was evaluated by the percentage of CD47‐positive events and their MFI, compared with unstained or uncoated controls. To assess long‐term stability, iRGD‐functionalized mRBC@FOLactis were prepared using 5‐FAM–labeled iRGD, incubated in 10% serum at 37 °C for up to 15 days, and analyzed at Days 1, 7, and 15. Dual‐color staining with AF647‐anti‐CD47 identified bacteria retaining both RBC membranes and iRGD as dual‐positive (FAM⁺/AF647⁺). The proportion and fluorescence intensity of dual‐positive cells were compared across time points. Appropriate controls, including single‐stain and FMO samples, were included to ensure gating accuracy and signal specificity.

### iRGD Peptide Functionalization

To further enhance tumor targeting and penetration, the tumor‐penetrating peptide iRGD was conjugated to the surface of mRBC@FOLactis. A bifunctional cell‐penetrating peptide (CPP) linker was used to facilitate the covalent attachment of iRGD onto the RBC membrane. The lyophilized iRGD‐CPP peptide was reconstituted in sterile saline to form a working solution. Approximately 1 × 10^9^ CFU of mRBC@FOLactis in PBS were mixed with the iRGD‐CPP solution and incubated at room temperature for 1 h. After incubation, the mixture was centrifuged and washed with PBS to remove unbound peptide. The final formulation, designated as iRGD‐mRBC@FOLactis, was then collected for subsequent in vitro and in vivo experiments.

### Characterization of iRGD‐mRBC@FOLactis

Engineered bacterial formulations were systematically characterized using a suite of analytical techniques to evaluate the impact of RBC membrane coating and subsequent iRGD functionalization. DLS was employed to measure both the hydrodynamic diameter and ζ potential of uncoated FOLactis, mRBC@FOLactis, and iRGD‐mRBC@FOLactis. DLS measurements were performed at a controlled temperature (25 °C) and under standardized conditions, ensuring reproducibility through appropriate sample dilution and fixed scattering angle settings.

To confirm the efficacy of the coating process, confocal fluorescence microscopy was used. In this assay, bacterial cells were pre‐stained with DAPI to label intracellular nucleic acids (green), while the RBC membranes were labeled with DiI (red). Merged images were subsequently analyzed for colocalization of the fluorescent signals using image analysis software. TEM was also conducted on negatively stained samples to observe the continuity and integrity of the membrane layer enveloping each bacterium.

Furthermore, bacterial viability post‐coating was assessed by CFU assays on MRS agar. These assays were carried out in triplicate under standardized conditions to determine any adverse effects of the RBC membrane coating process on bacterial survival and proliferation.

### Macrophage and DC Uptake Assay

An in vitro phagocytosis assay was conducted to evaluate the impact of RBC membrane camouflage on bacterial uptake by immune cells. Murine RAW264.7 macrophages and bone marrow–derived DCs were cultured in glass‐bottom dishes and coincubated with GFP‐expressing FOLactis (uncoated) or mRBC@FOLactis at 37 °C for 6 h. After incubation, the cells were carefully washed with phosphate‐buffered saline (PBS) to remove any extracellular bacteria. High‐resolution images were then obtained using confocal fluorescence microscopy under standardized settings. The resulting images were examined to assess the extent of bacterial internalization, thereby verifying the improved immune evasion conferred by the RBC membrane coating.

### 5.1 Tumor Spheroid Penetration and Cytotoxicity Assays

Three‐dimensional tumor spheroids were established by seeding approximately 5 × 10^3^ A549 cells per well in ultralow attachment plates. The cells were cultured under standard conditions for several days until the spheroids reached a diameter of about 500 µm. After spheroid formation, a 24‐h treatment was performed with one of four conditions: PBS (control), free iRGD, unmodified mRBC@FOLactis, or iRGD‐mRBC@FOLactis. Following the treatment period, GFP fluorescence imaging was conducted using confocal microscopy with Z‐stack acquisition. In this step, standardized imaging parameters—including laser intensity, z‐step intervals, and exposure time—were set to assess the penetration depth and spatial distribution of the bacteria within the spheroids. In parallel, a live/dead assay was performed by staining with Calcein‐AM (to mark live cells) and propidium iodide (to mark dead cells), with all assay conditions strictly controlled to ensure reproducibility.

### Animal Models and Treatment Protocols

For the subcutaneous tumor model, 1 × 10^6^ LLC cells suspended in 100 µL PBS were injected subcutaneously into the right flank of C57BL/6 mice. Once tumor volume reached approximately 80–100 mm^3^ (usually around 7 days post‐inoculation), mice were randomly assigned to four treatment groups: (1) NS (control), (2) mRBC alone, (3) uncoated FOLactis, and (4) mRBC@FOLactis. Treatments were administered via tail vein injection on Days 7, 14, 21, and 28. Tumor dimensions were measured every 3–4 days with calipers, and tumor volume was calculated using the formula (length × width^2^)/2.

For the orthotopic lung tumor model, approximately 5 × 10^5^ LLC cells in 50 µL PBS were injected intrapulmonary or via the tail vein to establish lung tumors. One week after tumor establishment, mice were randomized into four groups: (1) NS control, (2) anti‐PD‐1 antibody alone, (3) iRGD‐mRBC@FOLactis alone, and (4) combination of anti‐PD‐1 antibody with iRGD‐mRBC@FOLactis. The bacterial formulations were administered at 1 × 10^9^ CFU per injection via the tail vein, while the anti‐PD‐1 antibody was given at a dose of 200 µg per mouse. Throughout the experiment, body weight and tumor volume were monitored. Mice were euthanized when tumor volume reached 1500 mm^3^, and tissues were collected for biodistribution and immunohistochemical analyses. Survival data were analyzed using Kaplan–Meier curves with log‐rank tests.

### ELISA

ELISAs were performed to measure Flt3L and OX40L levels in vivo. Tumor‐bearing mice treated with iRGD‐mRBC@FOLactis or control groups were sacrificed at predefined time points (Days 1, 3, 7, 10, and 15). Blood samples were collected by cardiac puncture and centrifuged to obtain serum. Tumors and TDLN were excised, weighed, and homogenized in cold PBS containing protease inhibitors, followed by centrifugation to collect supernatants. The concentrations of Flt3L and OX40L in serum, tumor lysates, and TDLN lysates were quantified using commercial mouse ELISA kits (Flt3L: KE10072; OX40L: CSB‐EL023994MO) according to the manufacturers’ instructions. Absorbance was read at 450 nm with a microplate reader, and cytokine concentrations were calculated from standard curves. The results were expressed as pg per mL of serum or pg per mg of tissue protein and compared across groups and time points to assess the temporal dynamics of cytokine release.

### In Vivo Biodistribution and Imaging

Biodistribution studies were conducted using both noninvasive in vivo imaging and ex vivo organ analyses. LLC cells stably expressing firefly luciferase (LLC‐Luc) were implanted to form tumors. On Days 7, 14, 21, and 28 after implantation, mice received an intraperitoneal injection of D‐luciferin (150 mg kg^−1^), and imaging was subsequently performed using an IVIS Spectrum system under standardized conditions to capture the photon flux from the tumor regions.

In the bacterial distribution experiment, mice were administered DiR‐labeled mRBC@FOLactis or iRGD‐mRBC@FOLactis via tail vein injection. Near‐infrared fluorescence imaging was performed at predetermined time points (2 h, 24 h, and 3 days post‐injection) to monitor the distribution and accumulation of the bacteria within the lung tumors. After the final imaging session, major organs—including the lungs, liver, spleen, and kidneys—were harvested. These organs were then subjected to ex vivo fluorescence imaging and bacterial colony counting on MRS agar plates to quantitatively assess the in vivo distribution of the formulations.

### Flow Cytometry Analysis of Tumor Immune Microenvironment

Tumor tissues and other specimens were harvested under sterile conditions and mechanically dissociated using surgical scissors. The resulting tissue fragments were enzymatically digested with collagenase IV (1 mg mL^−1^) and DNase I (0.1 mg mL^−1^) at 37 °C for 30 min. The cell suspension was then filtered through a 70‐µm nylon strainer to remove undigested debris, and RBCs were subsequently eliminated using an ammonium chloride lysis buffer.

Before staining, cells were incubated with Zombie NIR viability dye (BioLegend, USA) according to the manufacturer's instructions for 20 min at room temperature in the dark, followed by washing with PBS. Surface staining was performed using the following fluorochrome‐conjugated antibodies at optimized concentrations: antibodies to CD45 (BV510, BD), CD3 (FITC, BD), CD4 (BUV395, BD), CD8α (PerCP‐Cy5.5, BD), CD25 (APC, BioLegend), Foxp3 (PE, eBioscience), CD69 (BV605, BioLegend), CD44 (Alexa Fluor 700, BD), CD62L (PE/Cyanine7, BioLegend), IFN‐γ (BV786, Invitrogen), IL‐4 (PE‐CF594, BioLegend), IL‐17A (BV421, BioLegend), CD11c (BV421, BioLegend), I‐A/I‐E (MHC II, FITC/PE, BioLegend), CD103 (PE/Cy7, BioLegend), CD11b (BV605, BD), CD80 (APC, BioLegend), and CD86 (PE, BioLegend). For intracellular staining of Foxp3, cells were fixed and permeabilized using a commercial kit per the manufacturer's instructions and then stained with the corresponding antibody.

Data acquisition was carried out on a BD LSRFortessa flow cytometer with standardized compensation settings and control samples, ensuring a minimum of 100000 events per sample. The gating strategy involved excluding debris and doublets using forward and side scatter parameters, identifying viable cells by excluding Zombie NIR‐positive cells, and subsequently gating on the viable CD45+ immune cell population. Within this population, T‐cell subpopulations (CD3+CD4+ and CD3+CD8+ cells) and DC subsets (CD11c+MHC‐II+ cells) were further delineated, with additional analysis of activation markers (CD69, CD44, CD62L, IFN‐γ, IL‐4, and IL‐17A) and maturation markers (CD80 and CD86). Data analysis was performed using FlowJo software to achieve detailed immune profiling.

### Multiplex Immunohistochemistry (mIHC)

Multiplex immunohistochemistry based on tyramide signal amplification (TSA) was performed to visualize CD4⁺, CD8⁺, and CD11c⁺ cells in tumor sections. Formalin‐fixed, paraffin‐embedded tissues were deparaffinized, rehydrated, and subjected to heat‐induced antigen retrieval. Iterative staining cycles were then performed using validated antibodies (CST) against CD4, CD8α, and CD11c, each followed by HRP‐conjugated secondary antibodies and distinct TSA fluorophores (Opal 520, 570, and 650). Between cycles, heat treatment was applied to remove bound antibodies while preserving deposited signals. Nuclei were counterstained with DAPI. Slides were scanned on a Vectra Polaris multispectral imaging system, and cell densities were quantified using inForm 2.6 software.

### Statistical Analysis

Statistical analyses were carried out with GraphPad Prism 9.0 (GraphPad Software, San Diego, USA). Before formal testing, all datasets were checked for normal distribution and homogeneity of variance. Outliers were only excluded when they could be clearly attributed to a technical error. Unless otherwise specified, continuous variables are expressed as mean ± SD. For immunophenotyping experiments, data are presented as mean ± SEM to better reflect variation within immune cell subsets. Sample sizes (n) for each experiment are provided in the figure legends.

For comparisons between two groups, two‐tailed unpaired Student's *t*‐tests were used when the data met assumptions of normality and equal variance. When more than two groups were compared, one‐way or two‐way ANOVA was performed, followed by appropriate post‐hoc tests (Tukey's or Bonferroni correction) to adjust for multiple comparisons. If normality or equal variance assumptions were not satisfied, nonparametric alternatives were applied (Mann–Whitney *U* test for two groups, Kruskal–Wallis with Dunn's post‐hoc test for multiple groups). Survival curves were analyzed using the Kaplan–Meier method, and differences between groups were evaluated by the log‐rank (Mantel–Cox) test. A *p* value of ≤ 0.05 was considered statistically significant.

## Conflict of Interest

The authors declare no conflict of interest.

## Supporting information



Supporting Information

## Data Availability

The data that support the findings of this study are available from the corresponding author upon reasonable request.
