# Supplementary material for: Intravenous iRGD‐Guided, RBC‐Membrane Camouflaged Lactococcus Lactis Remodels Cold NSCLC and Enhances PD‐1 Blockade
Source: Adv Sci (Weinh). 2025 Oct 3;12(43):e09604. doi: 10.1002/advs.202509604 (PMC12631879; doi:10.1002/advs.202509604)
Supplement: Supplementary file 1 — Supporting Information [file ADVS-12-e09604-s001.docx]

**
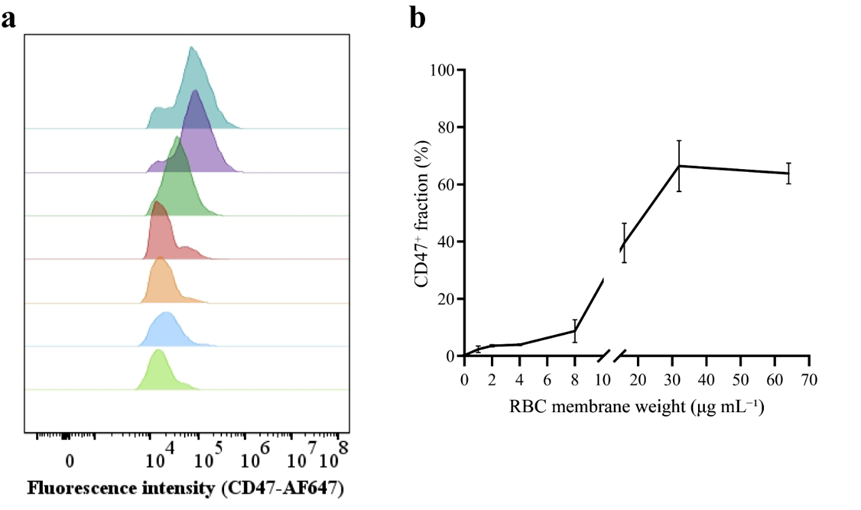
**

**Supplementary Figure 1. Flow cytometric quantification of RBC membrane coating efficiency using CD47.**

a.Representative flow cytometry histograms of CD47-AF647 fluorescence intensity on FOLactis coated with different amounts of RBC membrane vesicles (0–64 μg mL⁻¹).

b.Quantitative analysis of the CD47⁺ fraction (%) as a function of RBC membrane input, showing a dose-dependent increase and plateau at ~32 μg mL⁻¹. Data are presented as mean ± SD (n = 3 independent preparations). Statistical differences were analyzed using one-way ANOVA with Tukey’s post-hoc test. *p < 0.05, **p < 0.01.


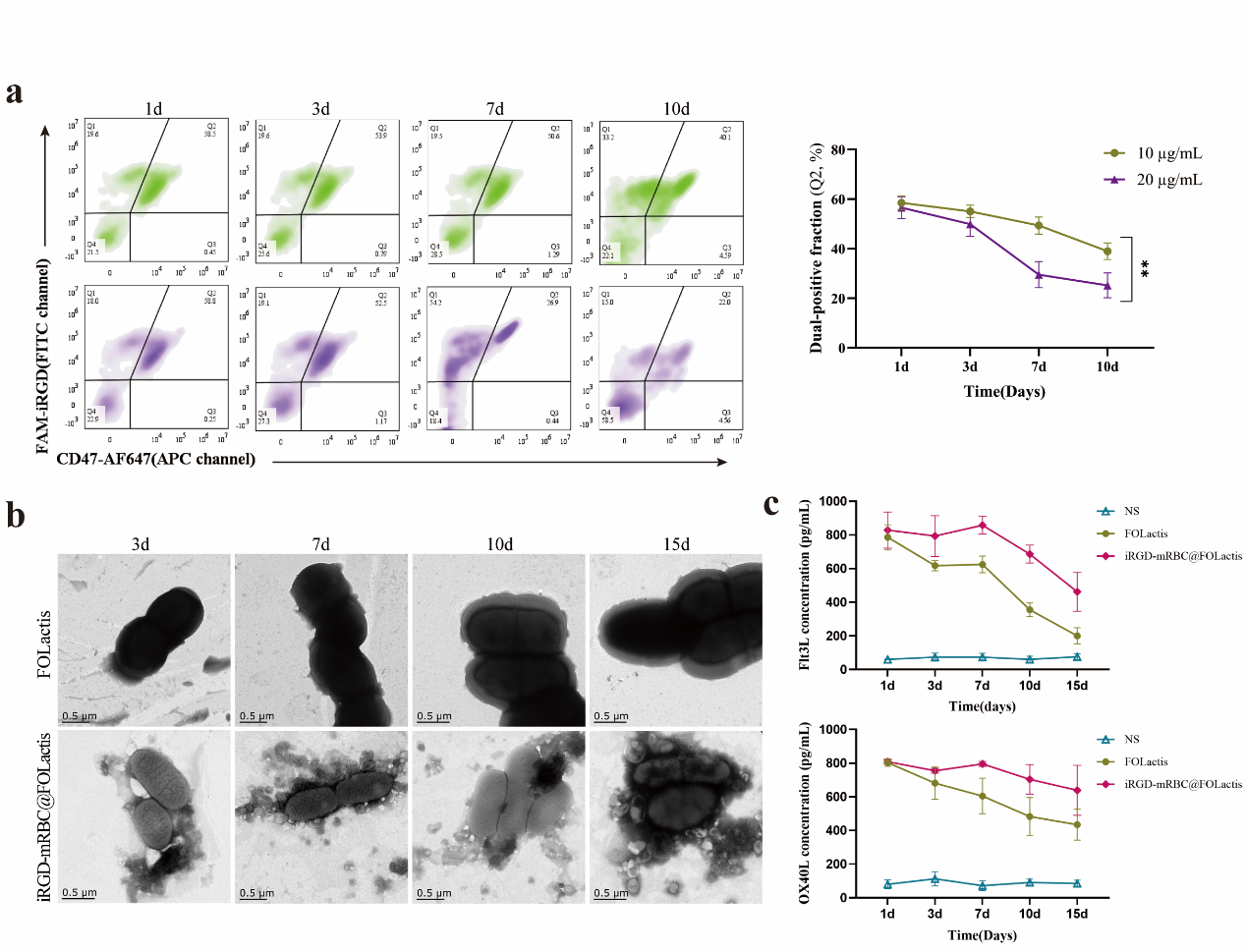


**Supplementary Figure 2. Long-term stability of iRGD-mRBC@FOLactis under serum stress.**

a.Dual-color flow cytometry (CD47–AF647 vs FAM-iRGD) showing the proportion of dual-positive bacteria at 1, 3, 7, and 10 days, indicating gradual decline but sustained stability under serum incubation.

b. Representative TEM images of iRGD-mRBC@FOLactis at 3, 7, 10, and 15 days, demonstrating preserved membrane coating and morphology.

c. ELISA quantification of Flt3L and OX40L secretion in tumors and draining lymph nodes at 1–15 days post-treatment, showing functional retention over time. Data are presented as mean ± SD (n = 3 independent preparations). Statistical differences were analyzed using one-way ANOVA with Tukey’s post-hoc test. *p < 0.05, **p < 0.01.


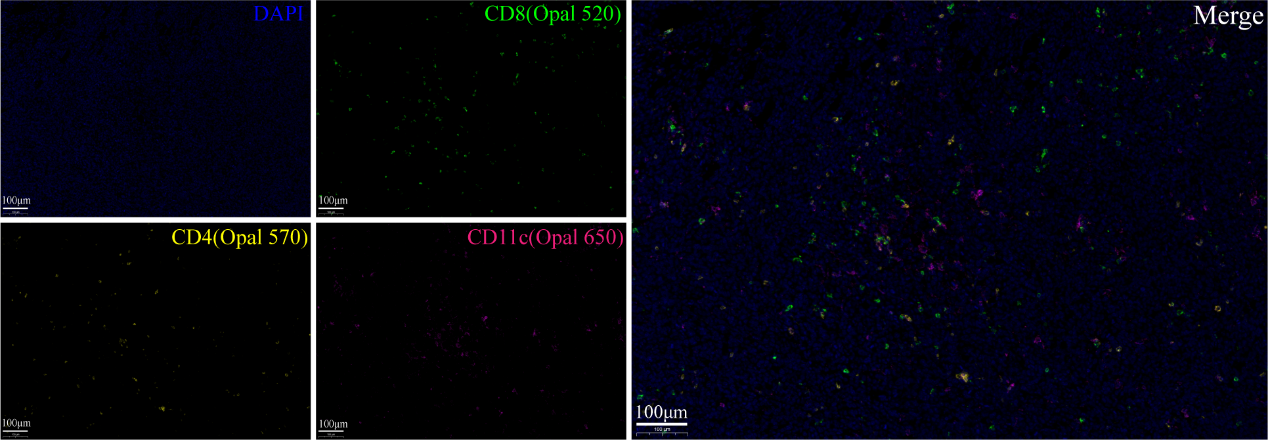


**Supplementary Figure 3. Single-channel validation of multiplex immunofluorescence staining.**

Representative tumor sections stained for CD8⁺ T cells (Opal 520, green), CD4⁺ T cells (Opal 570, yellow), and CD11c⁺ dendritic cells (Opal 650, red), with nuclei counterstained by DAPI (blue). Individual channels and the corresponding merged image are shown to verify signal specificity and separation. Scale bar, 100 μm. Related to Figure 8c–d.
